# Quantifying Soft Tissue Artefacts and Imaging Variability in Motion Capture of the Fingers

**DOI:** 10.1007/s10439-020-02476-2

**Published:** 2020-02-19

**Authors:** C. D. Metcalf, C. Phillips, A. Forrester, J. Glodowski, K. Simpson, C. Everitt, A. Darekar, L. King, D. Warwick, A. S. Dickinson

**Affiliations:** 1 Faculty of Environmental & Life Sciences, University of Southampton, Southampton, UK; 2 Faculty of Engineering and Physical Sciences, University of Southampton, Southampton, UK; 3 University Hospital Southampton, Southampton, UK

**Keywords:** Hand, Biomechanical modelling, MoCap, STA, Musculoskeletal, Kinematic, CT, Skin movement artefact

## Abstract

This study assessed the accuracy of marker-based kinematic analysis of the fingers, considering soft tissue artefacts (STA) and marker imaging uncertainty. We collected CT images of the hand from healthy volunteers with fingers in full extension, mid- and full-flexion, including motion capture markers. Bones and markers were segmented and meshed. The bone meshes for each volunteer’s scans were aligned using the proximal phalanx to study the proximal interphalangeal joint (PIP), and using the middle phalanx to study the distal interphalangeal joint (DIP). The angle changes between positions were extracted. The HAWK protocol was used to calculate PIP and DIP joint flexion angles in each position based on the marker centroids. Finally the marker locations were ‘corrected’ relative to the underlying bones, and the flexion angles recalculated. Static and dynamic marker imaging uncertainty was evaluated using a wand. A strong positive correlation was observed between marker- and CT-based joint angle changes with 0.980 and 0.892 regression slopes for PIP and DIP, respectively, and Root Mean Squared Errors below 4°. Notably for the PIP joint, correlation was worsened by STA correction. The 95% imaging uncertainty interval was < ± 1° for joints, and < ± 0.25 mm for segment lengths. In summary, the HAWK marker set’s accuracy was characterised for finger joint flexion angle changes in a small group of healthy individuals and static poses, and was found to benefit from skin movements during flexion.

## Introduction

Arthritis and inflammatory disease of the hand and wrist can have a devastating impact upon an individual’s quality of life by inhibiting simple, everyday tasks including personal care, preparing food and remunerative work. Surgical treatments for advanced degeneration of the finger joints (metacarpophalangeal (MCP), proximal interphalangeal (PIP) and distal interphalangeal (DIP)) include arthrodesis and arthroplasty. Joint replacement aims to reduce pain and maintain or restore function, but shows a complication rate in over 1/4 of replacements.^1^ Enhancing biomechanical measurement and modelling of the finger joints will in turn enable advances in joint replacement surgery, by improving implant design and surgical insertion.

A key biomechanical tool for hand and wrist functional analysis is computational musculoskeletal (MSK) modelling. These models are informed by incorporating several biomechanical data subsets, such as kinematics, electromyography and imaging. Ideally, multiple participants contribute to the dataset to make it anatomically and kinematically representative. Despite early attempts,^39^ standardisation of measurement techniques are yet to be widely adopted in the upper limb leading to alternative methods available to inform MSK models.^14^ Kinematic measurement techniques are often elected as the sole data source to inform the movement parameters of a MSK model, and differ from hand pose estimation, which is more commonly applied with markerless motion capture systems currently being widely adopted for applications in gesture-controlled interfaces.^16^

Marker-based kinematic measurement^11^,^27^,^28^,^32^,^34^ and MSK modelling techniques have been reported for the hand joints.^3^,^19^,^29^,^37^ The cited studies demonstrate that these methods are capable of reliable analysis of functional hand and wrist joint movements in health and disease, and integration into a MSK model can be used to predict kinetics such as joint moments,^5^,^31^ muscle forces in static tasks,^29^ and give insights into hand movements from extrinsic muscles with applications in prosthetic hand control.^3^ They are often limited by idealising anatomy and its variability and by available input data, requiring a compromise between the detailed measurements available from cadaver models and the representativeness of living human subjects. Moreover, where MSK models are driven by kinematic data, it is acknowledged that uncertainty arises from soft tissue artefacts (STAs), where motion capture markers display displacement relative to the underlying bony landmarks they are intended to follow.^8^,^40^ Additional challenges are faced in hand and wrist motion capture where the marker size is small compared to those used to analyse lower limb kinematics, and where the joint displacements and the anatomy itself are small and may occlude the markers. In hand and wrist analysis, Ryu *et al*. studied STAs at the MCP joint using magnetic resonance imaging^33^ and found a range of marker displacement distance relative to the second metacarpal, but a common displacement direction, which suggests these STAs may be corrected.

Prior research has also characterised the synchronous shifting of an entire marker set in unison (arising e.g. from inertial movement and underlying muscle activation), and relative marker displacements (arising e.g. from soft tissue deformations due to the underlying anatomy and apparent displacement from measurement inaccuracies) by comparing movements of skin- and skeletally-mounted markers.^4^,^14^,^36^ Methods exist to characterise STAs primarily in the larger joints and segments of the lower limb^7^ and the upper limb.^2^ Optimisation methods are established for calculating joint angles whilst accommodating the effects of STA, for example by minimising the difference between marker positions measured by motion capture and determined by a corresponding multi-link model with assumed joint constraints.^25^ Prior work has also been presented on how to reduce STAs at the knee by strategic selection of marker locations^12^ and how different marker placement protocols and topologies affect the different optimisation methods employed to accommodate STAs in joint angle estimation.^38^

The Hand and Wrist Kinematics (HAWK) measurement technique and associated single surface marker placement protocol was first reported in 2008.^28^ Its repeatability (inter-rater reliability) and accuracy were demonstrated, compared with static rigs. An enhanced version of the technique capable of describing pathological joint motion was also shown to be valid vs. static reference frames, with absolute joint flexion-extension angle error below 3.7°.^27^ We hypothesised, however, that accuracy might be improved both through STA correction and comparison with ‘gold standard’ skeletal kinematic measurements obtained from CT. Furthermore we set out to characterise the errors in joint angle arising from uncertainty in capturing the markers’ locations. We proposed the use of a rigid wand representing an extended finger and bearing markers, for which any changes in joint angle during imaging would represent error associated with measuring marker location.

Therefore, the objectives of this study were (i) to characterise the soft tissue artefacts through the range of motion of the PIP and DIP finger joints, (ii) to evaluate how correcting these artefacts influences the joint kinematic measurement accuracy using the HAWK measurement technique, and (iii) to determine the error associated with the motion capture system using markers mounted on a rigid, custom wand representing the third finger.

## Materials and Methods

### Participants

Between February 2015 and September 2017, ten participants were recruited for finger motion capture and imaging, according to an approved protocol (IRAS Ethics Ref: 14/LO/1059). These participants were free from hand or wrist disease or injury. They provided informed, written consent. Five participants of each sex were recruited (5F:5M) with an average age of 31 years (range 27–37 years).

### Data Collection

**Figure 1:**
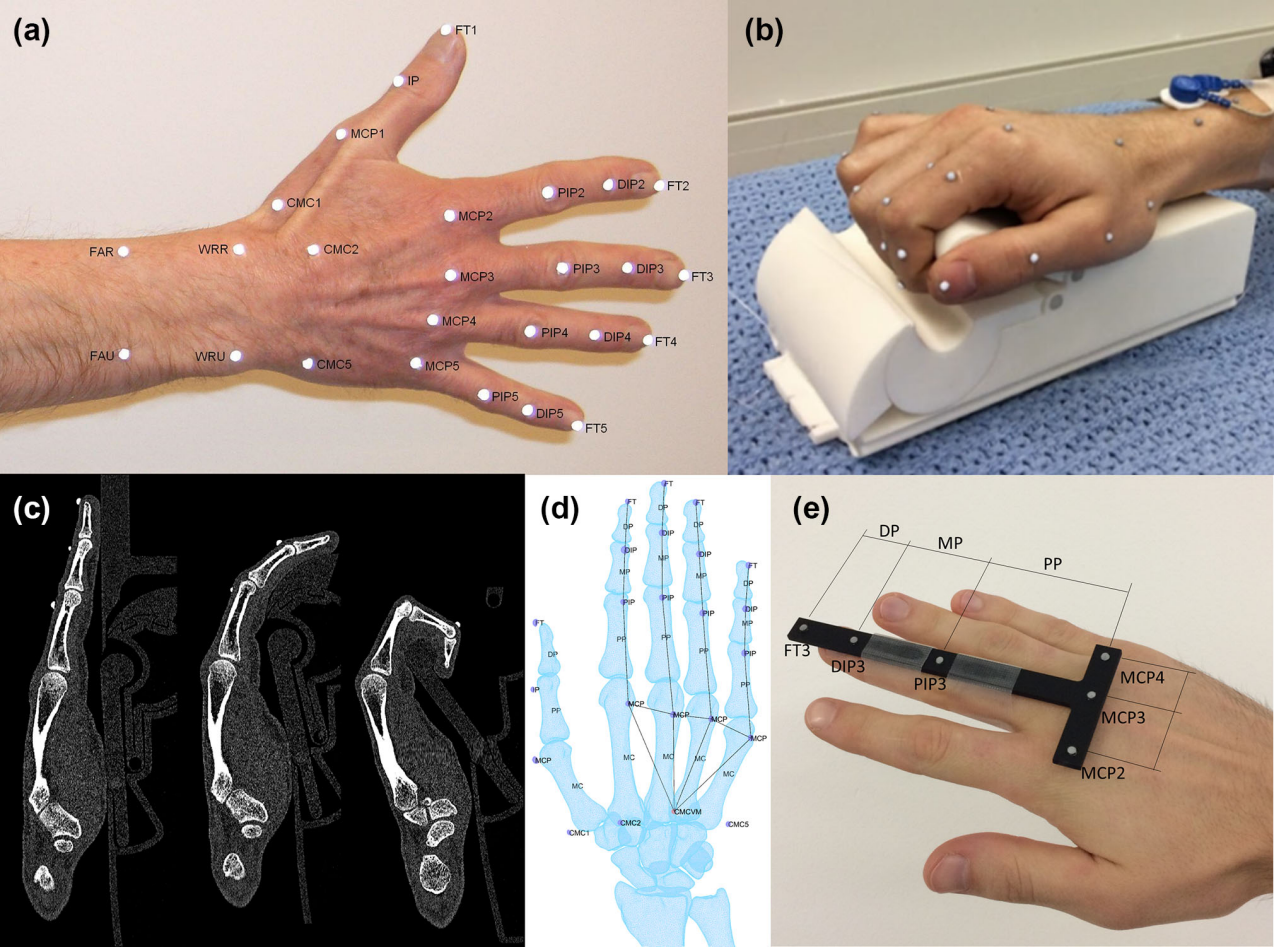
(a) HAWK 26-marker motion capture set, (b) finger pose support jig and (c) exemplar CT sections in three scanned positions. Note that markers are visible in CT data. (d) extracted bone and marker meshes, showing distal- and palmar-direction movement of the PIP marker relative to the reference proximal phalanx bone as joint flexion increases. (e) wand for system noise measurement, indicating ‘joint’ markers and finger segments.

The HAWK 26-marker-set^27^ was applied to the right hand of each participant by the same investigator (CDM), using 3 mm hemispherical reflective markers (Fig. 1a). Marker positions were captured by a Vicon T-Series system (6 × T160, 6 × T40) sampling at 100Hz. Each participant’s hand was then CT scanned (University Hospital Southampton Radiology department, Discovery CT750 HD 128 scanner, GE Healthcare Inc., USA) with near-isotropic voxel size (0.293 × 0.293 × 0.312 mm), 1825 ms exposure time, 0.7 mm spot size. Three scans were collected with the hand supported by an adjustable Nylon jig (Fig. 1b), with the fingers in full extension, mid flexion and near-full flexion (Fig. 1c).

### Data Processing

Reference joint angles were calculated by aligning each participants’ CT datasets in a functional coordinate system, and extracting the bones’ angular displacements. These were compared with joint angles calculated using the HAWK measurement technique which uses a global or laboratory coordinate system.

CT data were reconstructed and processed using ScanIP (Synopsys Inc., USA). Outlines of bones and markers were identified using a greyscale threshold, and the bodies were separated using a particle split function and each assigned a triangular surface mesh, stored in .stl format, where $B$ and $M$ are matrices of Cartesian coordinates of the bone and marker mesh vertices, respectively (Fig. 1d). These were imported into a MATLAB environment.

For each marker$M$ and joint $J$ a functional local coordinate system was constructed. The following description uses a PIP marker and joint example with the proximal phalanx (PP) as the proximal ‘reference’ bone and the middle phalanx (MP) as the distal bone; the same principle applies for the MCP joint with the MC and PP bones, respectively, and the DIP joint with the MP and DP bones, respectively.

**Figure 2:**
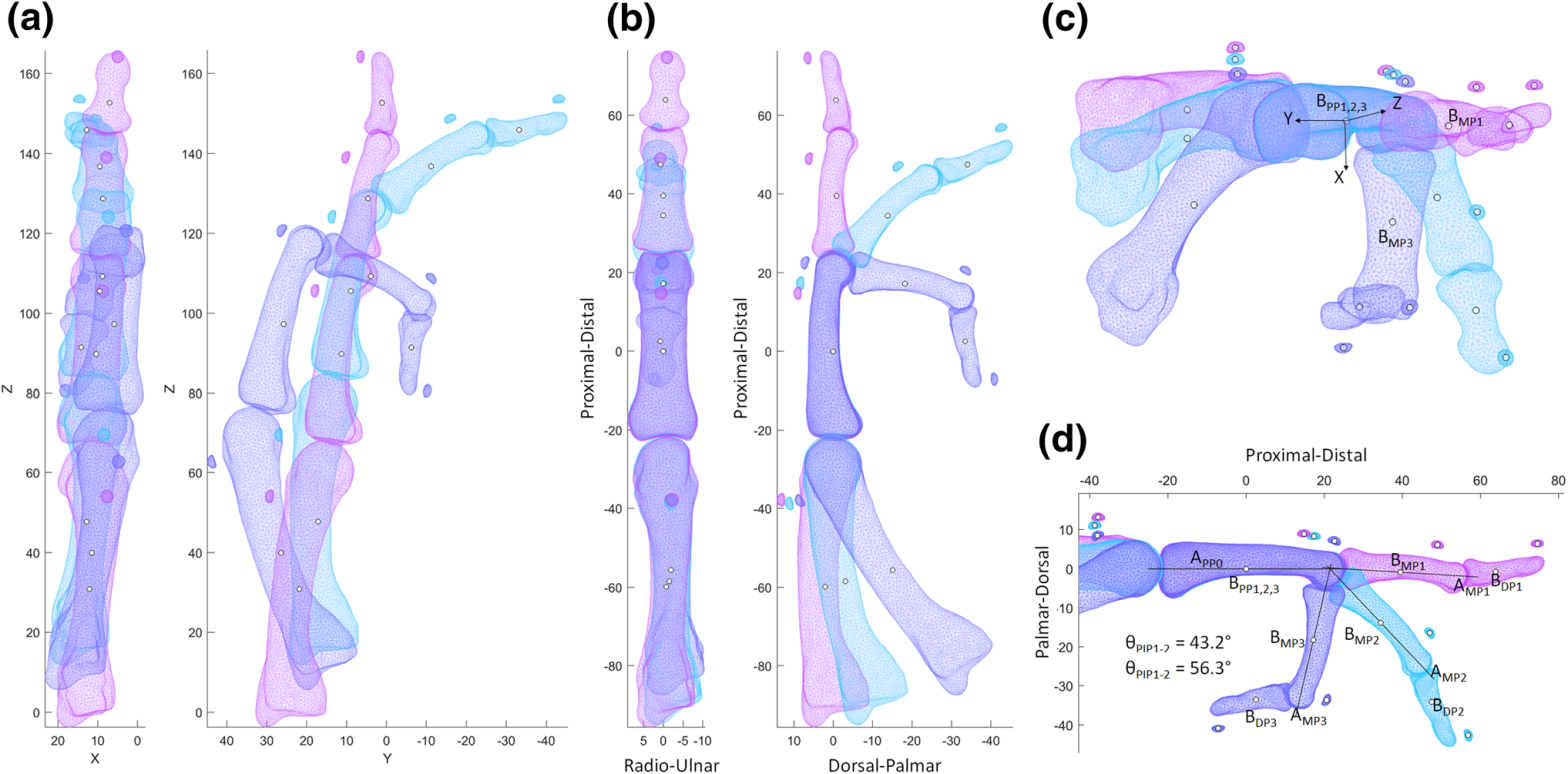
Aligning each group of three scans into a functional PIP coordinate system: (a) raw data; (b) PP surface centroids aligned at global origin, PP surfaces matched, and PP principal axis aligned with global z-axis; and (c) all data rotated into a functional coordinate system, for (d) exemplar anatomic joint angle measurement.

A unit vector representing the long axis of each bone (e.g. $\vec{A}_{{{\text{PP}}1}}$, for the proximal phalanx in scan position 1) was estimated by performing principal component analysis (PCA) upon its surface mesh vertices.^33^ A temporary local coordinate system origin was then defined as the centroid of the PP mesh vertices in position 1, $\bar{B}_{{{\text{PP}}1}}$, and a Rodriguez rotation was used to rotate all bones from the scan position 1 dataset so that the first principal direction of the PP surface $\vec{A}_{{{\text{PP}}1}}$ aligned in the sagittal plane with the local coordinate system’s y-axis. The full set of meshes from scan positions 2 and 3 were then moved into the reference coordinate system by aligning the reference bones (PPs) using iterative closest point matching (ICP). The local coordinate system was then updated to account for the joint’s function. A vector from $\bar{B}_{{{\text{PP}}0}}$ to $\bar{B}_{{{\text{MP}}1}}$, the MP bone surface centroid in full extension, was then aligned in the palmar-distal plane with the y-axis. Finally, a vector from $\bar{B}_{{{\text{PP}}0}}$ to $\bar{B}_{{{\text{MP}}3}}$, the MP bone surface centroid in near-full flexion, was aligned in the proximal-distal plane with the x-axis. This is illustrated for the PIP joint example (Fig. 2), and the reference bone principal axis is hereafter referred to as $\vec{A}_{{{\text{PP}}0}}$.

Thus, the angular change between each pair of scan positions for each distal bone gave the joint flexion-extension angle. As the bone principal axes were expressed as unit vectors:

$$\theta_{{{\text{PIP}}1 - 2}} = \cos^{ - 1} \left( {\vec{A}_{{{\text{MP}}1}} \cdot \vec{A}_{{{\text{MP}}2}} } \right)$$

Addressing Aim 1, the raw marker locations were extracted using the marker mesh vertex centroids, for the three scan datasets aligned by the joint’s proximal bone. The soft tissue movement from the fully extended scan to the mid and full flexion scans (as seen in Fig. 1d) was calculated as the change in marker surface centroid location in the reference bone coordinate system, along- and transverse to the reference bone axis, e.g.:

$$\vec{M}_{{{\text{PIP}}1 - 2}} = \overline{M}_{{{\text{PIP}}2}} - \overline{M}_{{{\text{PIP}}1}}$$

To address Aim 2, these marker locations were then corrected (i.e. the marker vertices moved to the same position relative to the joint’s proximal bone as in the reference, fully extended CT scan) for each individual. The HAWK method was then used to calculate the flexion angle of each joint based on the raw and corrected marker locations. This computes a resultant joint angle using two vectors normal to planes representing the proximal and distal segments for each joint. The segment planes are defined using the corresponding finger joint markers and the metacarpal joint markers of the neighbouring finger. The joint’s angle is calculated by the scalar product of the two normal vectors projected onto a plane orthogonal to the two segment planes. A direction multiplier is then used to identify whether the joint is in flexion or hyperextension, using the intersection of the normal vectors. This technique is equivalent to the CT-based technique which used vectors representing the joint segments themselves and a functional coordinate system defined by the plane in which maximal extension and flexion occurred. Agreement between the joint angles obtained from CT and marker data was tested by linear regression and Bland Altman analysis.

Addressing Aim 3 (motion capture system uncertainty), in a single participant, an aluminium reference T-shaped wand was precision manufactured (Fig. 1e) to represent the third finger and neighbouring MCP joints. Markers were added representing MCP2, MCP3, MCP4, PIP3, DIP3 & FT3. Assuming that the wand was a rigid segment, variation in the calculated angles would represent the imaging system uncertainty. The same 3 mm hemispherical markers were mounted on the face of the wand using Araldite 2-part epoxy adhesive (Huntsman Advanced Materials (Switzerland) GmbH). The positions of the markers on the wand were captured using the following three methods:

1. A static capture placed on a flat surface; and attached to the participant’s hand:
2. A dynamic capture for translations of the pseudo-fingertip marker across *X*, *Y*, *Z* axes relative to a defined global coordinate system; and
3. A dynamic capture for rotations around *X*, *Y*, *Z* axes.

The dynamic movements were approximately 400 mm/s (translations) and 400 deg/s (rotations), which corresponded to the maximum FT marker speed in a range of motion test. These speeds were guided via real-time visual feedback from the marker trajectory projected on a screen in front of the participant during capture. Again the PIP and DIP joint angles were calculated using the HAWK protocol, and the standard deviation of joint angles varying over time were used to describe the motion capture imaging uncertainty by calculating a 95% confidence interval (± 1.96 × s.d.).

## Results

The calculated soft tissue artefact displacements of the PIP and DIP joint markers showed a systematic movement with joint flexion, along the bone in a distal direction and slightly towards the bone axis in a palmar direction (Table 1). There was a small marker movement in the ulnar direction at the PIP joint, and no significant radio-ulnar marker movement at the DIP joint.

**Table 1:** Soft tissue artefact displacements for PIP and DIP joints across whole cohort. N.B. DIP marker was occluded for one participant

Soft tissue artefact	Displacement	Spearman *r*	*p*	*m* (mm/°)	*c* (mm)	RMSE (mm)
PIP	Resultant	0.889	< 0.001	0.053	0.0	0.894
	Distal	0.877	< 0.001	0.048	0.0	0.977
	Palmar	0.815	< 0.001	0.017	0.0	0.371
	Radial	− 0.441	< 0.001	− 0.006	0.0	0.631
DIP	Resultant	0.915	< 0.001	0.055	0.0	0.590
	Distal	0.907	< 0.001	0.051	0.0	0.612
	Palmar	0.888	< 0.001	0.015	0.0	0.229
	Radial	0.052	0.661	0.003	0.0	0.414

Linear regression expressed by Displacement = *m* * Angle + *c*

**Figure 3:**
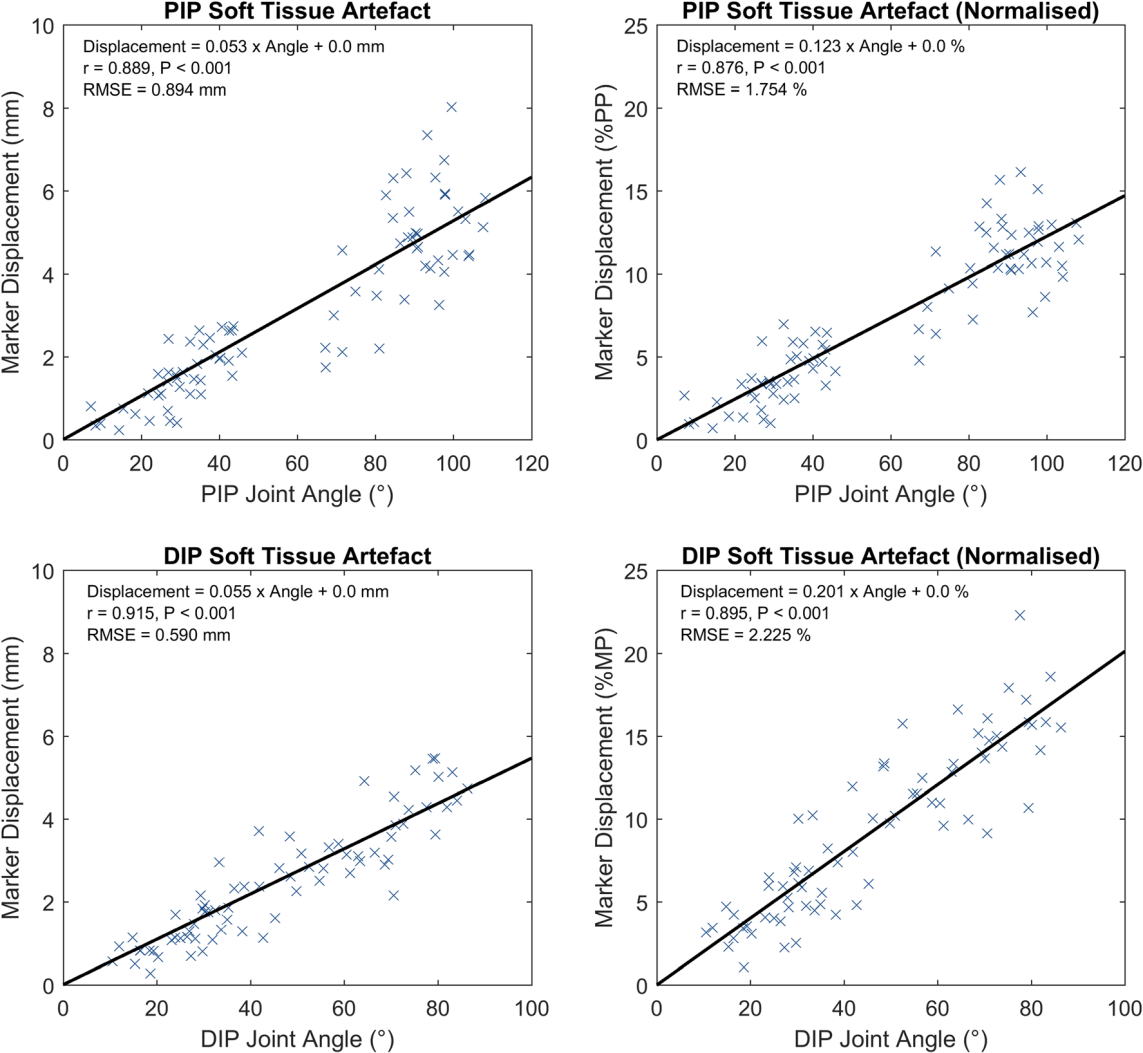
Resultant soft tissue artefact displacements for PIP (top) and DIP (bottom) joints across whole cohort, raw (left) and normalised to the joint’s proximal bone length (right). N.B. DIP marker was occluded for one participant.

The resultant soft tissue artefact displacements (i.e. total distance) of the PIP and DIP joint markers were plotted (Fig. 3), both raw and expressed as a percentage of the proximal bone of each joint (the proximal phalanx and the middle phalanx, respectively). A fitted linear regression indicated that both PIP and DIP joint markers typically moved approximately 0.55 mm for each 10° of flexion.

**Figure 4:**
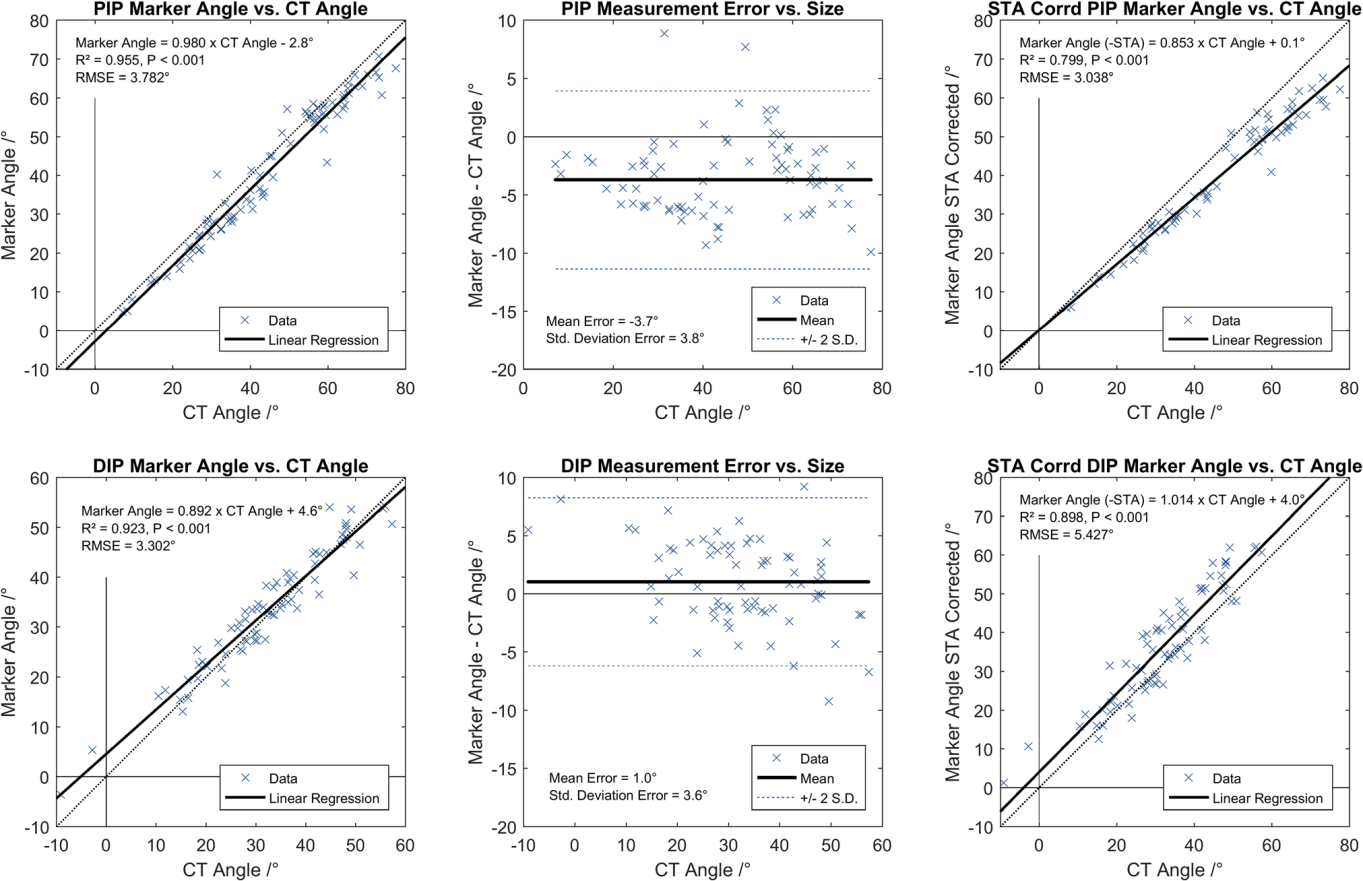
Correlation of PIP (top) and DIP (bottom) joint range of motion (ROM) obtained from bone principal axes (‘CT’) and marker vectors (left). Bland Altman (middle) plots show accuracy and precision of measurements. Correcting marker locations by removing soft tissue artefacts (right) worsened the marker-CT correlation. N.B. DIP marker was occluded for one participant

**Figure 5:**
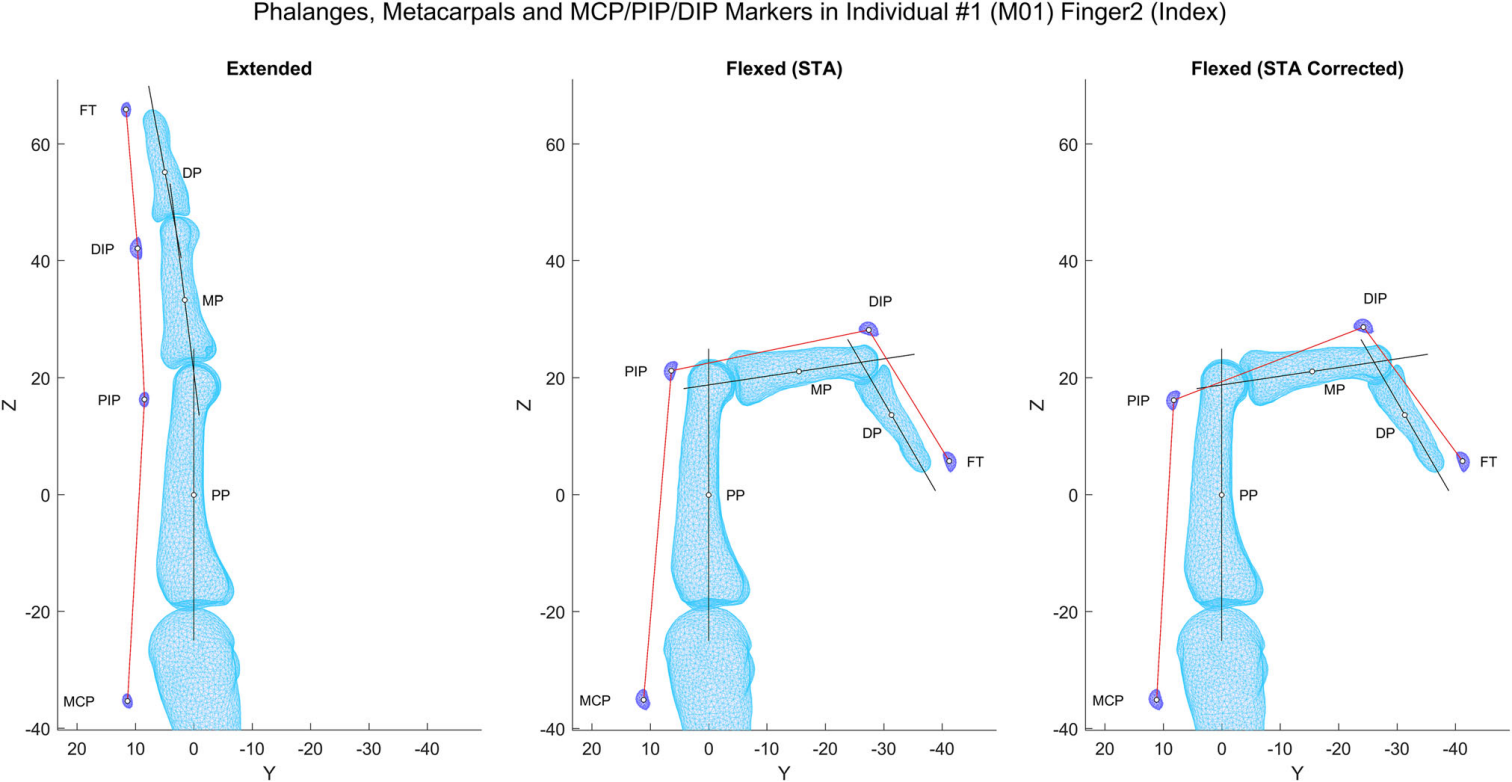
Illustration of the influence of correcting soft tissue artefacts upon marker vector representation of PIP and DIP joint angles. Note increased marker vector—bone axis misalignment particularly for middle and distal phalanges.

Table 2 shows the correlation between HAWK (marker position) and CT (underlying anatomy) joint range of motion for PIP and DIP joints for all participants across all fingers, and per finger. A significant linear regression was observed for the PIP joints (top) and DIP joints (bottom) for all participants (Fig. 4 left). Bland Altman analysis shows that the PIP marker gave a small systematic underestimate of the PIP angle (− 3.7°), and high precision (95% confidence interval < 4°). DIP angle change measurement was accurate to within 1°, but the deviation between CT and marker angle increased with measurement size (Fig. 4 middle). Correcting marker displacements due to soft tissue artefact (Fig. 4 right, Fig. 5) weakened the correlation at the PIP joint by considering both regression slope (*m*) and coefficient of determination (*R*^2^). The correlation gradient was improved at the DIP joint by correcting marker displacements, although the scatter (RMSE) was increased vs. measurements obtained using the raw marker vectors.

**Table 2:** Statistics of correlation between HAWK (marker-based) and CT (anatomic) joint range of motion (ROM) for PIP and DIP joints across whole cohort, in all cases and per-finger

HAWK:CT correlation	Finger(s)	*R*^2^	*p*	*m* (°/°)	*c* (°)	RMSE (°)
PIP	All (*n* = 80)	0.955	< 0.001	0.980	− 2.782	3.782
	2 (*n* = 20)	0.945	< 0.001	0.948	− 0.592	3.575
	3 (*n* = 20)	0.907	< 0.001	1.017	− 4.195	4.098
	4 (*n* = 20)	0.969	< 0.001	1.038	− 5.978	2.955
	5 (*n* = 20)	0.970	< 0.001	0.953	− 2.541	3.940
DIP	All (*n* = 72)	0.923	< 0.001	0.892	4.554	3.302
	2 (*n* = 18)	0.928	< 0.001	0.927	3.036	2.494
	3 (*n* = 18)	0.940	< 0.001	0.801	7.266	2.945
	4 (*n* = 18)	0.949	< 0.001	0.836	6.865	2.584
	5 (*n* = 18)	0.928	< 0.001	1.051	− 0.588	3.822

*N.B. DIP marker was occluded for one participant. Linear regression expressed by HAWK Angle = *m* CT Angle + *c*

**Figure 6:**
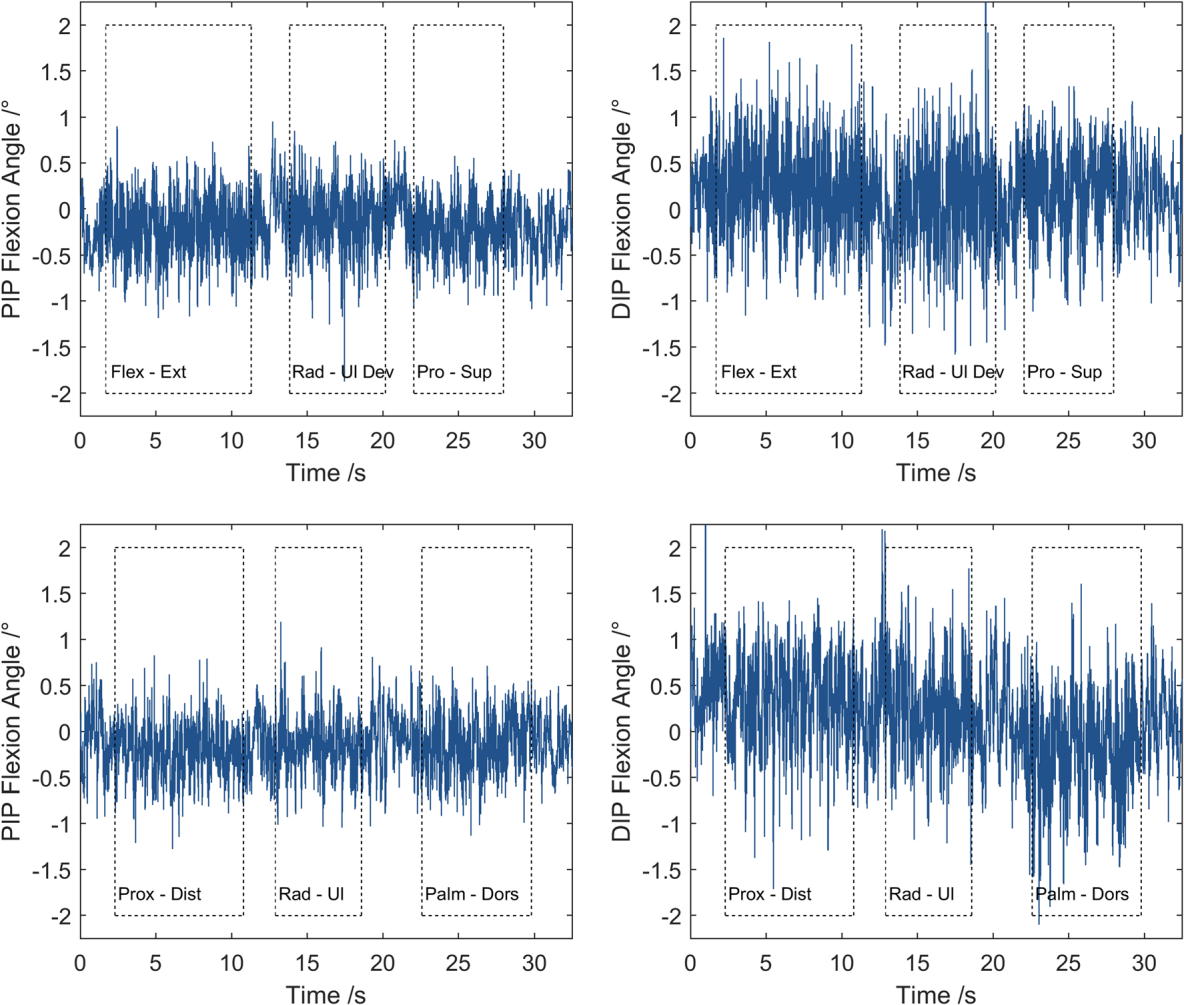
Example flexion angles at PIP (left) and DIP (right) ‘joints’ on wand during motion capture representative-speed 3-axis rotations (top) and translations (bottom). Periods of movement indicated by boxes.

The results of the wand mounted statically or moving with representative-speed 3-axis translations and rotations show the system variability in PIP and DIP ‘joint’ flexion angles, and PP and MP ‘segment’ lengths (Fig. 6, Table 3). The 95% confidence interval was smaller than ± 1° for angles in both joints, and smaller than ± 0.25 mm for segment lengths.

**Table 3:** Bias and sensitivity (mean and standard deviation) of PIP and DIP ‘joint’ angles, and proximal phalanx and mid phalanx segment lengths, during motion capture of wand using static and representative-speed 3-axis translations and rotations.

		PIP	DIP
Joint F–E angle Mean (S.D.) (°)	Static	− 0.02 (0.10)	− 0.31 (0.20)
	Translations	− 0.23 (0.31)	0.32 (0.46)
	Rotations	− 0.11 (0.31)	0.15 (0.47)

		Proximal phalanx	Middle phalanx
Segment length Mean (S.D.) (mm)	Static	60.12 (0.03)	37.21 (0.03)
	Translations	59.87 (0.13)	37.33 (0.08)
	Rotations	59.89 (0.11)	37.24 (0.09)

## Discussion

This study provides data on the accuracy of skin-mounted passive marker placement in calculating finger joint movement. Specifically, our data compare the position of the marker with respect to the underlying anatomy derived from CT scans from ten healthy participants. The importance of determining marker displacement due to STA or system error is established. The former is relevant to the rigid body assumption routinely used during rotation and translation of body segments in kinematic analysis of human movement^15^,^20^ and the latter determines the reconstruction error from the motion capture system.

Our results show that a systematic displacement occurs relative to the underlying anatomy in the PIP and DIP joints using the HAWK single surface marker placement protocol. This displacement was observed to maintain the marker—bone axis vector alignment through the joint’s range of motion. Inspection of a typical example illustrates that as the PIP and DIP joints flex, the soft tissue artefact causes the PIP and DIP markers to translate along the proximal and middle phalanges, respectively, toward the joints’ instantaneous axes of rotation (Fig. 5). This cam-follower effect gives smaller misalignment between the marker vectors and corresponding bone axes than would occur if the markers remained static with respect to the underlying bone. For the HAWK marker set, this indicates a systematic soft tissue displacement artefact occurs, which is beneficial to the accuracy of marker range of motion measurement, and dominates more stochastic artefacts such as inertial movement.

These findings support and extend previous results summarised in the review by Lee and Jung,^24^ who recommended a single surface marker placement protocol is sufficient for capturing static positions. They also recommended using three markers per segment, or cluster marker sets, for dynamic motion capture as these protocols are less affected by skin movement. The current study’s findings expand on that previous work by providing evidence to the effect of skin movement using a single surface marker placement protocol, against a CT imaging reference.

Previous research in hand and wrist kinematic measurement studied STA at the MCP joint using magnetic resonance imaging^33^ and found a marker displacement distance ranging from 1.16 to 10.86 mm relative to the second metacarpal while held in a fixed position during flexion of the fingers. The authors noted a common displacement direction across participants. Buczek at al.^5^ also found a marker displacement of approximately 10 mm across the MCP joints but commented that this did not affect the phalanges. In contrast, the results from this study establish and quantify the error from marker displacement affecting the phalanges, in terms of the arising finger joint angle measurements. A similar CT-based method was presented by Buffi *et al*.^6^ for assessing the accuracy and precision of finger joint angle measurements in an instrumented glove, for a single individual. In the present study, the PIP joint had a smaller absolute uncertainty than DIP for angle calculations, possibly because the PIP joint total marker distance (MCP-PIP-DIP) is larger than the DIP joint total marker distance (PIP-DIP-FT), for which the same marker positional uncertainty would be expected to produce a larger angular uncertainty.

The effect of STA is reported to be compounded by the application of, and measurement from, skin surface markers,^21^ and arguably a similar assertion could be made for technical marker sets or clusters placed on frames and then on the skin surface. The additional distributed mass of frame-mounted clusters might produce increased non-systematic STA, especially in dynamic conditions. Similar results were reported by Nataraj and Li.^31^ Other researchers have investigated methods of defining local frames of reference for hand kinematic measurement, and acknowledge the need for validation against bone movements from medical imaging.^18^ Alternative validation approaches include goniometry^13^ and planar fluoroscopy, applied to the hand^10^ and the foot.^35^

This study is limited by the small sample size. Nevertheless, we provide data that could be used to support future researchers in calculating their required sample size to ensure statistical power and evaluating potential clinical effect sizes, and offer insights to support previous work undertaken in cadaveric or single case studies. These results would complement advanced soft tissue MSK models,^3^,^17^,^23^,^26^,^29^,^30^ now finding use in such diverse applications as studying human-device interactions^22^ and optimising tenodesis surgery.^9^ These results also quantified the error associated with STA affecting and advocating a single surface marker placement protocol on the fingers as an alternative approach to technical marker sets or marker clusters, which has been an ongoing topic of debate in kinematic analysis studies. An additional error, not discussed in this study, is that associated with palpation and marker placement (see Ref. 28 for a discussion relevant to hand biomechanics). Reliability was not addressed here primarily because repeat CT scans could not be conducted ethically.

Our study demonstrates a systematic soft tissue movement for motion capture markers on the fingers and that a single surface marker placement protocol provides an assessment of underlying anatomy, now with a known accuracy. These results can be used to justify the use of reduced marker sets, which may be more appropriate in hand biomechanics where available skin surface is extremely limited and more complex marker clusters can interfere with, or alter, the assessment of functional movements.
